# Body size ideals and body satisfaction among Dutch-origin and African-origin residents of Amsterdam: The HELIUS study

**DOI:** 10.1371/journal.pone.0252054

**Published:** 2021-05-26

**Authors:** Jody C. Hoenink, Henrike Galenkamp, Erik J. Beune, Marieke A. Hartman, Marieke B. Snijder, Henriette Dijkshoorn, Ron J. G. Peters, Ellen Bal, Karien Stronks, Mary Nicolaou

**Affiliations:** 1 Department of Epidemiology and Biostatistics, Amsterdam Public Health Research Institute, Amsterdam UMC, Vrije Universiteit Amsterdam, Amsterdam, the Netherlands; 2 Department of Public Health, Amsterdam Public Health Research Institute, Amsterdam UMC, University of Amsterdam, Amsterdam, the Netherlands; 3 Department of Life Sciences, Erasmus University College, Erasmus University Rotterdam, Rotterdam, the Netherlands; 4 Department of Clinical Epidemiology, Biostatistics and Bioinformatics, Amsterdam Public Health Research Institute, Amsterdam UMC, University of Amsterdam, Amsterdam, the Netherlands; 5 Department of Epidemiology, Health Promotion & Healthcare Innovation, Public Health Service of Amsterdam, Amsterdam, the Netherlands; 6 Department of Cardiology, Amsterdam UMC, University of Amsterdam, Amsterdam, the Netherlands; 7 Department of Social and Cultural Anthropology, VU University, Amsterdam, the Netherlands; University of Mississippi Medical Center, UNITED STATES

## Abstract

**Objective:**

Obesity is highly prevalent among ethnic minorities and acceptance of larger body sizes may put these ethnic minorities at risk of obesity. This study aimed to examine body size ideals and body satisfaction in relation to body weight, in two Sub-Saharan African (SSA)-origin groups in the Netherlands compared to the Dutch. Additionally, in the two SSA-origin groups, this study assessed the mediating role of acculturation in the relation between ethnicity and body size ideals and body satisfaction.

**Methods:**

Dutch, African Surinamese and Ghanaians living in Amsterdam, the Netherlands, participated in the observational HELIUS study (n = 10,854). Body size ideals were assessed using a validated nine figure scale. Body satisfaction was calculated as the concordance of current with ideal figure. Acculturation was only assessed among SSA-origin participants and acculturation proxies included age of migration, residence duration, ethnic identity and social network. Weight and height were measured using standardised protocols.

**Results:**

SSA-origin women and Ghanaian men had larger body size ideals compared to the Dutch; e.g. Surinamese and Ghanaian women had 0.37 (95%CI 0.32; 0.43) and 0.70 (95%CI 0.63; 0.78) larger body size ideals compared to Dutch women. SSA-origin participants were more often satisfied with their weight compared to the Dutch. Similarly, SSA-origin participants had more than twice the odds of being satisfied/preferring a larger figure compared to the Dutch (e.g. B_Surinamese men_ 2.44, 95%CI 1.99; 2.99). Within the two SSA-origin groups, most acculturation proxies mediated the relation between ethnicity and body size ideals in women. Limited evidence of mediation was found for the outcome body satisfaction.

**Conclusion:**

Public health strategies promoting a healthy weight may need to be differentiated according to sex and ethnic differences in body weight perception. Factors other than acculturation may underlie the ethnic differences between African Surinamese and Ghanaians in obesity.

## Introduction

Overweight and obesity is highly prevalent among migrants living in high-income countries, including African-origin populations [1, 2], which form an increasingly large population group in Europe [3]. For example, a literature review investigating the prevalence of cardiovascular disease, diabetes and risk factors among populations of sub-Saharan African (SSA) descent consistently found that a higher percentage of these migrants were classified as obese compared to the general population (e.g. 43% of African Surinamese women were classified as obese compared to 14% of Dutch women) [1].

Socio-cultural factors influence the standards of desirable body weight within cultures [4]. For example, in parts of SSA, overweight and obesity have traditionally been considered to be a sign of success, prosperity, good health and happiness [5, 6], although this view is reported to be changing [6, 7]. The acceptance of larger body sizes and body size ideals may put SSA-origin populations at risk of obesity [8]. Presumably, accurate perception of overweight status may be needed to motivate healthy behaviours [9]. Therefore, understanding body weight perception is needed for the development of public health strategies to address the high obesity prevalence in SSA-origin populations.

Body weight perceptions of SSA-origin populations living in Africa may not be comparable to SSA-migrants living in high-income countries as body weight perceptions may change as a result of immigration and acculturation [4]. Because in many high-income countries slim figures are generally preferred [5], SSA-origin migrants, especially women, may prefer smaller body sizes compared to SSA populations living in Africa. Acculturation refers to the process of cultural exchange that develops when two cultures come into continuous first-hand contact. This can result in changes to cultural features, predominantly in the minority group, in various ways (e.g. by adopting or rejecting the receiving culture and retaining or rejecting the heritage culture) [10–12].

This acculturation process has indeed been found to influence body size ideals and body satisfaction in a number of studies [13–15]. It is therefore possible that migrants or migrant groups that are more exposed to the culture in high-income countries (i.e. more Western cultures) have smaller body ideals compared to migrant groups that have been less exposed to this culture. For example, a study found that SSA-migrant women living in the United Kingdom had a more negative body image perception and were more dissatisfied with their body compared to the SSA-origin women [16].

This study examined body size ideals and body satisfaction in two SSA-origin groups living in Amsterdam, the Netherlands, compared to the Dutch-origin population. Specifically, this study investigated; 1) the body size ideals of Dutch, African-Surinamese and Ghanaian origin men and women, 2) whether the two SSA-origin populations are more likely to be satisfied with their actual body size at a higher level of BMI than the Dutch and to assess whether this differs for persons with overweight and obesity, and 3) if potential differences in the body size ideals and body satisfaction between the two SSA-origin populations are explained by differences in acculturation levels. African-Surinamese and Ghanaians were included in the study as the two African-origin migrant groups have a different history of contact with the Dutch culture and, presumably, different levels of acculturation to the Dutch culture. The Surinamese originated from West Africa and were sent as slaves to Surinam between the 16^th^ and 19^th^ century. As a group, they have a long history of contact with the Dutch, speak Dutch and have been living in the Netherlands for many decades. Ghanaians are the largest SSA migrant group in the Netherlands with, on average, a shorter residence duration; many migrated in the beginning of the 1990’s. The hypothesis is that because the African-Surinamese have a longer history of contact with the Dutch culture compared to the Ghanaians, their body size ideals would be situated between those of the Dutch (smallest body size ideals) and Ghanaians (largest body size ideals). Any differences in body size ideals and body satisfaction between African-Surinamese and Ghanaians may be explained by their different acculturation levels.

## Material and methods

### Study design and population

For this study, Dutch, African-Surinamese and Ghanaian origin participants of the HELIUS study with complete data on body mass index (BMI) and body size ideal variables were selected. The HELIUS study is a cohort study among six ethnic groups (18–70 years), living in Amsterdam, the Netherlands [17]. The study design has been described previously [17]. Briefly, participants of the HELIUS study were randomly sampled, stratified by ethnicity, from the municipal register of Amsterdam. Of the 90 019 persons invited, baseline data were collected among 24 789 participants of varying ethnicities (response rate of 28%) [17]. Participants filled in a questionnaire and underwent a physical examination, including anthropometric measures. Written informed consent was obtained from all participants before starting the study [18]. The study complies with the Declaration of Helsinki and was approved by the Institutional Review Board of the Amsterdam Medical Centre.

### Ethnicity

The current study only included Dutch, African-Surinamese and Ghanaian participants. Only African-Surinamese and Ghanaian participants were included due to their shared ancestry, but different exposure to the Dutch culture. As described in the introduction, the two African-origin migrant groups have a different history of contact with the Dutch culture and, presumably, different levels of acculturation to the Dutch culture due to their different lengths of contact with the Dutch.

Ethnicity was defined using the country of birth, including the country of birth of both parents. More specifically, participants who were born in Surinam or Ghana and had at least one parent born there too, were considered to be of Surinamese or Ghanaian origin (first generation). Participants born in the Netherlands and with two parents born in Surinam or Ghana were considered to be second generation migrants. Participants were considered to be of Dutch origin if the person and both parents were born in the Netherlands. Because the Surinamese population mainly consists of five large ethnic groups, African-origin Surinamese (hereafter referred to as Surinamese) were included based on self-reported ethnic origin.

### Assessment of body size ideals and body satisfaction

During baseline data collection, body size ideals and body satisfaction were measured using the body image scale designed by Pulvers et al. [19]. The body image scale consisted of nine figures in a random order of smaller and larger figures. The figures were developed to resemble persons of multi-ethnic backgrounds, with attention to hair and facial features and to span BMIs of approximately 16 to 40 in increments of three BMI points [19].

Body size ideal was assessed by the question ‘Which figure would you most prefer to look like?’ with a possible score range of 1 to 9. Perception of current body size was determined by asking ‘Which figure do you most look like right now?’. Ethnic-specific mean BMI per figure was assigned based on the mean BMI of participants that chose that figure to represent their current body size. To investigate whether the three ethnic groups interpret the nine body figures equally, the BMIs associated with each of the figures in the scale per ethnic group were analysed.

Body size satisfaction was defined as the body size ideal in relation to the self-perceived body size. This was calculated by subtracting the self-perceived current body figure from the ideal body figure. Participants with a body discrepancy score of 0 were considered to be satisfied with their body. Participants with a positive body discrepancy score were considered dissatisfied and preferring a larger body figure, while participants with a negative body discrepancy score were considered dissatisfied because they prefer a smaller body figure. Subsequently, body size satisfaction was related to the actual BMI by assessing the average BMI score for satisfied and unsatisfied participants of the three ethnic groups.

### Assessment of acculturation proxies

Given the cross-sectional study design, all measures of acculturation are considered to be ‘proxies’ of acculturation. Acculturation was only measured in Surinamese and Ghanaian participants. The acculturation proxies were selected to represent three global thematic domains within acculturation research: migration history, ethnicity and social environment [20]. Two indicators of migration history were included–age of migration and residence duration. While these are not direct measures of acculturation they are commonly used proxies in the literature and have been found to perform well in relation to other acculturation measures [21]. Measures of social network were included as, based on literature, it seems that social comparison may be an important determinant of body size ideals and obesity [22, 23]. Social network was assessed using the statement ‘I have Dutch friends’ and ‘I spend most of my free time with Dutch people. Finally, we included an indicator of ethnic identity. Ethnic identity was assessed using the statement ‘I feel Dutch’. Each item was rated on a 5-point Likert scale. A higher score indicates higher acculturation to the Dutch culture.

### Other variables

Covariates were included based on evidence from the literature and included age, sex, educational level and BMI. Weight and height were measured in duplicate during the physical examination in barefoot subjects wearing light clothes only, conducted by trained research assistants. Participants with a BMI (kg/m^2^) of ≥ 25.0 were considered overweight and those with a BMI of ≥ 30.0 were considered obese. Educational level was based on self-report and assessed through the participant’s highest level of education and categorized into one of four categories: never been to school or elementary school only, lower vocational/secondary schooling, intermediate vocational/secondary schooling and higher vocational schooling or university.

### Data analyses

Statistical analysis was performed using IBM SPSS Statistics version 23.0 and R Studio, version 1.2.1335 on Windows [24]. All analyses were stratified on the basis of sex as there are considerable differences between men and women with regards to body image [25, 26]. Descriptive statistics for socio-demographic variables, acculturation proxies and the outcome variables by ethnicity and sex were reported using percentages, means and standard deviations or medians and interquartile distances in case of non-normality. To assess the calibration of the image scale, the mean BMI and corresponding SD were calculated for each figure representing participants current body, separately by ethnicity and sex. Linear regression analysis was used to determine ethnic differences in body size ideals. Furthermore, logistic regression analysis was used to examine ethnic differences in body satisfaction (0 = prefer smaller; 1 = satisfied/prefer larger) for those with a normal weight and overweight/obesity. Logistic regression analysis instead of ordinal regression analysis was used for the outcome body satisfaction given the low proportion of participants being unsatisfied and preferring a larger body figure, the better interpretability of the results and the possibility to assess mediation by acculturation. As it can be argued that participants who are satisfied should not be combined with participants who prefer to be larger, we conducted a sensitivity analysis (0 = prefer smaller; 1 = satisfied) to assess whether the addition of those preferring a larger body affected the results in the main analyses.

Mediation analyses were conducted on a subsample of the population, namely the African Surinamese and the Ghanaians, as it is not possible for the Dutch to acculturate to the Dutch population. The mediation package in R [27] was used to determine the mediating roles of the acculturation proxies. Residence duration and age of migration were only relevant for first generation migrants; the analyses of ethnic identity and social contacts included first and second generation participants. Mediation by acculturation proxies in the relation between ethnicity and the two outcome measures (i.e. body size ideals and body satisfaction) were tested separately for men and women. In total, this led to 16 mediation models. Mediation by acculturation was assessed in six steps with linear regression for continuous variables, and logistic regression for dichotomous variables. First, the associations between the independent variable ethnicity and the dependent variables body size ideals and body satisfaction (i.e. the total effect or c-path, consisting of two different dependent variables) were assessed. Second, the associations between the independent variable ethnicity and the potential mediators acculturation proxies (a-path, consisting of the four different acculturation proxies) were assessed. Third, the associations between the potential mediators (acculturation proxies) and the dependent variables body size ideals and body satisfaction, adjusted for ethnicity (b-path) were assessed. Fourth, the associations between ethnicity and the dependent variables body size ideals and body satisfaction, adjusted for each mediator (i.e. the direct effect or c’-path) were assessed. Fifth, to assess whether acculturation mediated the association between ethnicity and body size ideals or body satisfaction, a bootstrapping procedure was conducted around the indirect effect (i.e. the a-path * b-path). The indirect effect was assessed with the mediate function, using results from the regression models for the a- and b-paths [27]. Last, as recommended by Hayes [28], the proportion mediated (indirect effect divided by total effect) was calculated, but only if *1)* significant mediation was found, *2)* the total (c path) and indirect (a-path * b-path) effect had the same direction and *3)* if the indirect effect was smaller than the total effect. S1 Fig displays the mediation model (S1 Fig).

All regression models included the covariates age and educational level. Statistical significance was determined if the upper and lower bound of the bias corrected 95% confidence intervals (CI) did not contain zero and complete case analysis was used.

## Results

### Baseline characteristics

Overall, n = 4531 Dutch, n = 4055 African-Surinamese and n = 2268 Ghanaian origin participants were included in the study. Mean age ranged from 43.3 (SD 10.8) to 48.1 (SD 12.9) (Table 1; Socio-demographic characteristics). Dutch-origin participants had the highest educational level and Ghanaian-origin participants the lowest. More African-origin participants, in particular women, belonged to the overweight/obese category as compared to Dutch-origin participant.

**Table 1:** Characteristics of the study population (N = 10,854) by sex and ethnic origin.

	Men			Women		
	Dutch (n = 2075)	Surinamese (n = 1579)	Ghanaian (n = 880)	Dutch (n = 2456)	Surinamese (n = 2476)	Ghanaian (n = 1388)
Socio-demographic characteristics						
Age in years, mean (SD)	46.9 (13.8)	48.1 (12.9)	46.8 (11.5)	45.5 (14.2)	47.8 (12.3)	43.3 (10.8)
Educational level^a^ (%)						
*1*. *(low)*	3.4	6.6	16.2	3.2	5.0	36.8
*2*.	13.6	40.8	45.6	14.7	32.6	36.4
*3*.	23.4	34.3	29.1	20.7	36.6	22.5
*4*. *(high)*	59.6	18.4	9.1	61.3	25.9	4.3
BMI Category (%)						
*<18*.*5*	0.6	1.9	0.5	2.7	1.5	0.9
*18*.*5–24*.*99*	52.7	39.5	35.3	63.2	27.1	17.5
*25–29*.*99*	36.3	41.2	46.6	24.0	33.8	37.2
*≥30*	10.2	17.4	17.6	10.1	37.5	44.5
Acculturation characteristics						
First generation migrant (%)	NA	83.2	95.3	NA	93.7	95.5
Residence Duration, mean (SD)^b^	NA	32.9 (10.6)	19.0 (8.1)	NA	31.5 (10.5)	17.8 (8.1)
Age of migration, mean (SD)^b^	NA	18.7 (10.2)	29.5 (8.7)	NA	19.7 (10.6)	27.2 (8.5)
Dutch ethnic identity score, mean (SD)^c^	NA	3.6 (1.2)	3.1 (1.1)	NA	3.5 (1.2)	3.0 (1.1)
Dutch social network score, mean (SD)^d^	NA	5.7 (2.0)	5.0 (1.8)	NA	5.7 (2.0)	4.6 (1.8)
Outcome variables						
Body size ideal, mean (SD)^e^	3.5 (0.0)	3.6 (0.0)	3.8 (0.0)	3.0 (0.0)	3.6 (0.0)	3.9 (0.0)
Body satisfaction (%)						
*Prefer smaller*	45.0%	34.2%	35.8%	61.0%	66.6%	60.0%
*Satisfied*	41.2%	50.2%	47.6%	36.0%	27.1%	30.9%
*Prefer larger*	13.8%	15.6%	16.6%	3.1%	6.3%	9.1%

^a^ 1 = “never been to school or elementary schooling only”, 2 = “lower vocational schooling or lower secondary schooling”, 3 = “intermediate vocational schooling or intermediate/higher secondary schooling”, and 4 = “higher vocational schooling or university”

^b^ Only in first generation migrants

^c^ Ranges from 1 through 5

^d^ Ranges from 1 through 10

^e^ Ranges from 1 through 9

Abbreviations: SD = Standard Deviation, NA = Not Applicable

Regarding the acculturation proxies, Surinamese participants had been living approximately 14 years longer in the Netherlands, and migrated to the Netherlands at a younger age, compared to Ghanaian participants (Table 1; Acculturation characteristics). Surinamese participants indicated that they relate more to the Dutch ethnic identity and have more Dutch social networks compared to Ghanaian participants.

The smallest average body size ideals were found for the Dutch and the largest were found for Ghanaians in both men and women (Table 1; Outcome variables). With regards to body satisfaction, most women preferred to have a smaller body size while most men were satisfied with their body with the exception of Dutch men.

### Body size ideals and body satisfaction

After adjusting for age and educational level, Ghanaian men had a larger body size ideal compared to Dutch men (B 0.15, 95%CI 0.08; 0.21), and there were no differences in body size ideals of Surinamese compared to Dutch men (B -0.02, 95%CI -0.07; 0.04). Ghanaian women had a 0.70 (95%CI 0.63; 0.78) and Surinamese women had a 0.37 (95%CI 0.32; 0.43) larger body size ideal compared to Dutch women.

**Fig 1:**
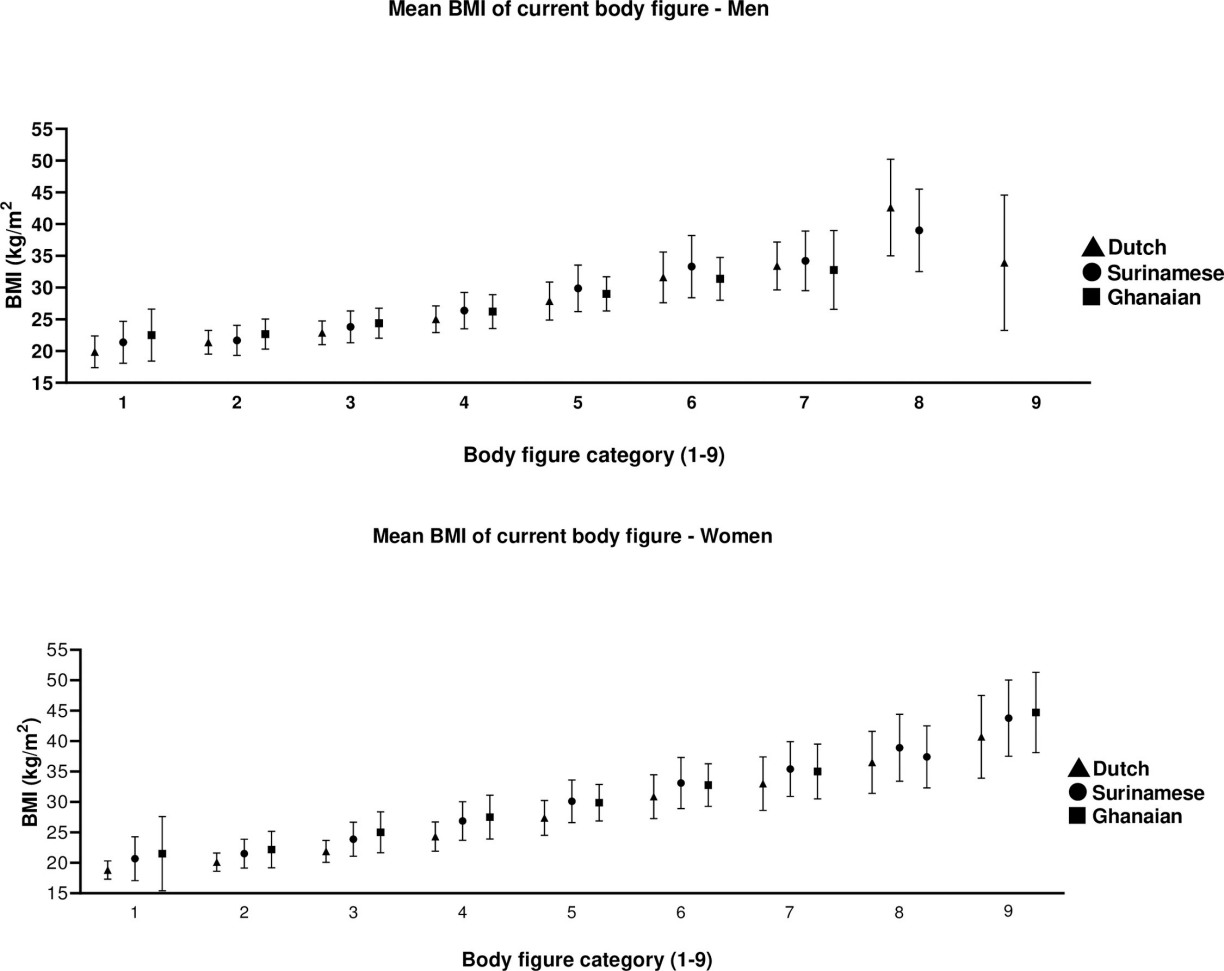
Mean BMI with corresponding 95% confidence interval associated with each of the body figures representing participants current body figure stratified by ethnicity for men and women.

Dutch participants generally had lower BMIs (but not statistically significantly given the overlapping 95% confidence intervals) when choosing a body figure that best represents their current body compared to African-origin participants (Fig 1 and S1 Table for a complete overview of these numbers). The average BMIs of the most chosen body Figs 3 and 4 were within or only slightly above a healthy BMI (Fig 4 represented BMIs between 24.3 and 27.5).

Within all three weight classifications, African-origin men were more satisfied with their body size or preferred a larger body size compared to Dutch-origin men (Table 2). For example, 30% of overweight Dutch men were satisfied with their body compared to 53% of overweight African-origin men. Overall, a larger proportion of Dutch men preferred to be smaller in all three weight classifications compared to African-origin men.

**Table 2:** Body satisfaction, by weight status, sex and ethnicity.

		Men				Women			
	BMI categories	Prefer smaller (%)	Satisfied (%)	Prefer larger (%)	N	Prefer smaller (%)	Satisfied (%)	Prefer larger (%)	N
Dutch	<18.5–24.99	21.2	54.5	24.3	1105	46.0	49.5	4.5	1619
	25–29.99	67.8	30.1	2.1	760	87.6	12.1	0.3^a^	589
	> = 30	88.1	11.4	0.5^1^	210	95.2	4.8	0.0^a^	248
	**Total**	**45.0**	**41.2**	**13.8**	**2075**	**61.0**	**36.0**	**3.1**	**2456**
Surinamese	<18.5–24.99	7.5	60.0	32.5	653	29.9	51.5	18.6	709
	25–29.99	41.6	53.5	4.9	651	71.0	26.3	2.7	838
	> = 30	80.0	19.3	0.7^1^	275	90.6	9.3	0.1^a^	929
	**Total**	**34.2**	**50.2**	**15.6**	**1579**	**66.6**	**27.1**	**6.3**	**2476**
Ghanaian	<18.5–24.99	8.6	54.9	36.5	315	13.3	54.1	32.5	255
	25–29.99	40.7	53.4	5.9	410	55.2	39.5	5.2	516
	> = 30	78.1	17.4	4.5^1^	155	83.3	14.1	2.6	617
	**Total**	**35.8**	**47.6**	**16.6**	**880**	**60.0**	**30.9**	**9.1**	**1388**

^a^ N<10

Abbreviations: BMI = Body Mass Index

Similar results were found for women (Table 2). A larger proportion of African-origin women classified in the overweight or obese category were satisfied with their body size compared to Dutch women. Furthermore, a larger proportion of African-origin women in the normal weight category preferred to be larger compared to Dutch women (i.e. 5% of Dutch, 19% of Surinamese and 33% of Ghanaian women). On the other hand, 46% of Dutch women categorized in the normal weight category would like to be smaller compared to 30% and 13% of Surinamese and Ghanaian women, respectively.

Irrespective of weight status, African-origin men and women had a higher odds of being satisfied or preferring a larger figure than Dutch-origin men and women (Table 3). For example, African-origin men with a normal weight had a 2.34 (95%CI 1.47; 3.71) and 2.96 (95%CI 2.07; 4.24) higher odds of being satisfied or preferring a larger body compared to Dutch men. African Surinamese women classified as having a normal weight and overweight/obesity had a 1.81 (95%CI 1.49; 2.21) and 2.01 (3.45; 7.90) higher odds of being satisfied or preferring a larger body compared to Dutch women, respectively. The odds of being satisfied or preferring a larger body were largest for Ghanaian women. The same results, with slightly smaller effect sizes, were found when comparing participants who were satisfied with their body to those who preferred to be smaller (S2 Table).

**Table 3:** Ethnic differences in body satisfaction by weight status.

	Satisfied or prefer larger vs. prefer smaller					
	Men			Women		
	Dutch	Surinamese	Ghanaian	Dutch	Surinamese	Ghanaian
BMI		OR (95%CI)	OR (95%CI)		OR (95%CI)	OR (95%CI)
<24.99	Ref	2.96 (2.07; 4.24)	2.34 (1.47; 3.71)	Ref	1.81 (1.49; 2.21)	5.22 (3.45; 7.90)
> = 25.00	Ref	2.40 (1.97; 2.94)	2.68 (2.11; 3.40)	Ref	2.01 (1.55; 2.62)	3.68 (2.74; 4.96)

Adjusted for age and educational level

Abbreviations: OR = Odds Ratio, 95%CI = 95% Confidence Interval, Ref = reference group

### Role of acculturation in body size ideals and body satisfaction

Amongst African Surinamese and Ghanaian men there was no indication of a mediating effect of acculturation on the association between ethnicity and body size ideal as well as body satisfaction (Tables 4 and 5; indirect effect). Amongst African Surinamese and Ghanaian women, three out of the four acculturation proxies significantly mediated the association between ethnicity and body size ideals (Table 4). The results indicate that the larger body size ideals in Ghanaian women (Table 4; total effect) is for a small part related to the fact that they are less acculturated to the Dutch culture compared to Surinamese women (Table 4; indirect effect). For the outcome body satisfaction, only ‘age of migration’ mediated the association between ethnicity and body size satisfaction (indirect effect Table 5: B 1.02, 95%CI 1.00–1.03).

**Table 4:** The mediating role of acculturation in the association between ethnicity and body size ideals for men and women separately^a^.

	Independent variable	Mediating variables	N	Effect of ethnicity on acculturation proxies (a-path)	Effect of acculturation proxies on body size ideals (b-path)	Direct effect (c’-path)	Indirect effect (a-path x b-path)	Total effect (c-path)	Proportion mediated
				B (95% CI)	B (95% CI)	B (95% CI)	B (95% CI)	B (95% CI)	$\frac{AB}{\left(C^{\prime }+AB\right)}$
Men	Ghanaian vs. Surinamese	Residence duration^b^	2081	-11.97 (-12.70;-11.24)	0.00 (-0.00;0.00)	0.19 (0.11;0.27)	-0.01 (-0.06;0.04)	0.18 (0.11;0.25)	NA
		Age of migration^b^	2081	12.02 (11.30;12.76)	-0.00 (-0.01;0.00)	0.19 (0.11;0.28)	-0.01 (-0.06;0.04)		NA
		Dutch ethnic identity	2412	-0.44 (-0.54;-0.35)	0.01 (-0.02;0.03)	0.17 (0.11;0.24)	-0.00 (-0.01;0.01)	0.17 (0.11;0.24)	NA
		Dutch social network	2422	-0.60 (-0.76;-0.44)	-0.00 (-0.02;0.01)	0.17 (0.11;0.24)	0.00 (-0.01;0.01)		NA
Women	Ghanaian vs. Surinamese	Residence duration^b^	3274	-10.19 (-10.90;-9.48)	-0.01 (-0.01;-0.00)	0.28 (0.19;0.38)	0.09 (0.04;0.13)	0.37 (0.28;0.46)	24.3
		Age of migration^b^	3274	10.23 (9.53;10.94)	0.01 (0.00;0.01)	0.28 (0.19;0.38)	0.09 (0.05;0.13)		24.3
		Dutch ethnic identity	3771	-0.45 (-0.54;-0.36)	-0.01 (-0.04;0.01)	0.34 (0.27;0.42)	0.01 (-0.01;0.02)	0.34 (0.27;0.42)	NA
		Dutch social network	3807	-0.72 (-0.87;-0.57)	-0.03 (-0.04;-0.01)	0.32 (0.25;0.40)	0.02 (0.01;0.03)		5.9

^a^ Analyses only include African Surinamese and Ghanaian participants as it is not possible for the Dutch to acculturate to the Dutch culture

^b^ Analyses include only first-generation migrants given that second-generation migrants (i.e., born in the Netherlands) do not have an age of migration or residence duration

Abbreviations; B = Beta regression coefficient, 95%CI = 95% Confidence Interval, NA = Not Applicable

**Table 5:** The mediating role of acculturation in the association between ethnicity and body satisfaction (satisfied/prefer larger vs. prefer smaller) in men and women with overweight or obesity^a^.

	Independent variable	Mediating variables	N	Effect of ethnicity on acculturation proxies	Effect of acculturation proxies on satisfaction	Direct effect (c’-path)	Indirect effect (a-path x b-path)	Total effect (c-path)	Proportion mediated^b^
				B (95% CI)	OR (95% CI)	OR (95% CI)	OR (95% CI)	OR (95% CI)	$\frac{AB}{\left(C^{\prime }+AB\right)}$
Men	Ghanaian vs. Surinamese	Residence duration^c^	1298	-11.5	0.99	1.01	1.02	1.03	NA
				-12.42;-10.58	0.98;1.01	0.94;1.08	0.98;1.06	(0.97;1.08)	
		Age of migration^c^	1298	11.57	1.01	1.01	1.02		NA
				10.65;12.49	0.99;1.02	0.94;1.08	0.98;1.06		
		Dutch ethnic identity	1464	-0.44	1.02	1.03	1	1.03	NA
				-0.56;-0.31	0.89;1.07	0.97;1.08	0.99;1.01	(0.98;1.08)	
		Dutch social network	1473	-0.54	1.01	1.03	1		NA
				-0.74;-0.33	0.95;1.06	0.98;1.09	0.99;1.01		
Women	Ghanaian vs. Surinamese	Residence duration^c^	2577	-10.13	0.99	1.06	1.02	1.07	NA
				-10.90;-9.36	0.98;1.00	1.03;1.08	1.00;1.03	(1.05;1.09)	
		Age of migration^c^	2577	10.16	1.01	1.06	1.02		28.6
				9.39;10.93	1.00;1.02	1.03;1.08	1.00;1.03		
		Dutch ethnic identity	2822	-0.42	0.98	1.07	1	1.07	NA
				-0.52;-0.31	0.91;1.06	1.06;1.09	1.00; 1.00	(1.06;1.09)	
		Dutch social network	2854	-0.74	0.96	1.07	1		NA
				-0.91;-0.58	0.91;1.01	1.05;1.09	1.00;1.01		

^a^ Analyses only include African Surinamese and Ghanaian participants as it is not possible for the Dutch to acculturate to the Dutch culture

^b^ Calculated based on the log transformed coefficients of the odds ratio (i.e. the Beta regression coefficient)

^c^Analyses include only first-generation migrants

Abbreviations; B = Beta regression coefficient, 95%CI = 95% Confidence Interval, OR = Odds Ratio, NA = Not Applicable

## Discussion

This study examined body size ideals and body satisfaction in two Sub-Saharan African-origin groups compared to the host population, and the potential mediating role of acculturation within the two Sub-Saharan African-origin groups. Our results are consistent with our hypothesis that Ghanaian women had larger body size ideals than Surinamese women and, in turn, Surinamese women had larger body size ideals than Dutch women. Furthermore, Ghanaian men had larger body size ideals compared to Dutch and Surinamese men. African-origin participants were more satisfied with their body size or preferred a larger body size compared to those of Dutch-origin. Among those with overweight or obesity, a significantly larger proportion of African-origin participants were satisfied with their body size compared to Dutch-origin participants. Evidence for a mediating role of acculturation in the association between ethnicity and body size ideals was found for women. There was hardly any evidence for a mediating role of acculturation in the association between ethnicity and body satisfaction.

On the one hand, African groups living in Europe may have similar body size ideals as African groups living in the United States (US) due to their shared ancestry. Contrarily, they may differ since African groups living in Europe are relatively recent migrants compared to African Americans. Nonetheless, many of our results are consistent with findings among African Americans [29–31]. For example, African-origin populations were more likely to prefer a larger figure compared to other ethnicities at the same BMI level [29, 32, 33]. Also, African-origin women were less dissatisfied with their body compared to white women [29, 33].

In our study, several acculturation proxies mediated the association between ethnicity and body size ideals in women. Furthermore, there was hardly any evidence that acculturation mediated the association between ethnicity and body satisfaction. As a strength of our study, to our knowledge, no studies have been conducted on this topic. Therefore, a comparison with previous literature regarding the mediating role of acculturation is limited. Most studies on acculturation investigated the association between acculturation and body perceptions among Hispanics living in the US [13, 34–36] and/or used different acculturation proxies (e.g. language, social networks and ethnic social relations) [20].

Previous studies have also noted sex differences in body perceptions [13, 25, 26, 37]. Similar to this study, smaller differences in body size ideals between white and African-Americans were found in men compared to women [25] and men were generally more satisfied with their body compared to women [26, 37]. A systematic review investigating the relation between acculturation and obesity among migrants in high-income countries found that the relationship between acculturation and obesity is less consistent in women than in men. For men, a predominantly positive association between acculturation and BMI was found, while for women both negative and positive associations were found [13].

The body size ideals of African-origin populations were expectedly larger than those of Dutch-origin, although the differences between men were less striking. Furthermore, even though the BMIs associated with the current figures were larger for African-origin participants compared to Dutch-origin participants, the figure chosen by the majority corresponded to BMIs within the higher end of a healthy weight range. It seems that public health strategies can promote body weights within the healthy BMI range as this would not contradict the body size ideals of these migrant groups. However, the value that African-origin populations place on achieving their ideal body size may be relatively low, since Surinamese and Ghanaian participants with overweight or obesity were more likely than Dutch participants to be satisfied with their current body weight. Therefore, public health strategies may need to emphasize other behavioural outcomes. For example, a qualitative study in Amsterdam indicated that African-origin groups may value good health above an ‘ideal’ body weight and that promoting health-enhancing behaviour such as physical activity may be perceived as a more positive health promotion message [38].

The majority of women indicated that they preferred a smaller body size, although this differed according to weight status. In the case of African-origin women, preferring a smaller size might reflect an appropriate level of dissatisfaction given that the majority of women were considered overweight or obese. The finding that between 40% and 90% of participants with overweight and obesity were dissatisfied and preferred a smaller body size may imply that they accurately perceived themselves as having overweight. However, dissatisfaction with one’s body size may have harmful effects on mental health [39]. Thus, strategies aimed to reduce overweight/obesity at the population level should promote health and well-being rather than thin bodies.

Another important finding is that Dutch women have ideals at the lower end of the healthy weight range; they chose between Figs 2 and 3, corresponding to a BMI of approximately 20.5 (or 21 according to the self-perceived figures). In addition, 46%, 30% and 13% of Dutch, Surinamese and Ghanaian women, respectively, in the healthy BMI range (18.5–25.0) indicated a preference for a smaller figure. These findings suggest that these women may be at risk of inappropriate dieting practices and, at the extreme, eating disorders [40, 41]. Thus, there is a need to promote more realistic, healthy body weight ideals amongst women, but especially amongst Dutch women.

There is a long history of contact between Suriname and the Netherlands, so the starting point of Surinamese participants regarding ‘acculturation’ may greatly differ from that of Ghanaians, which could explain why their body size ideals are smaller than those of the Ghanaians. Indeed, evidence for a role of acculturation in the body size ideals of women, but not men, was found. It seems that adopting a more Western culture has a greater impact on women’s body size ideals than on men’s. This is consistent with the idea that there is a greater focus on women’s body size/shape and, in high-income countries in particular, the promotion of a thin ideal [42].

No mediation by acculturation in the relation between ethnicity and body satisfaction was found. As previously mentioned, this may be explained by the value African-origin populations may place on achieving their ideal body size. Furthermore, despite their ties to the Netherlands and their long residence duration, there remain considerable differences between the Surinamese and the Dutch population. Contextual factors may explain these differences. For example, many African-origin residents of Amsterdam live in the ‘South East’ district, where 74% of all residents are of non-Dutch origin [43]. This undermines the underlying premise that acculturation is likely to result in ‘convergence’ of minority groups to a majority, mainstream, culture. Due to multiculturalism, there is not one ‘majority’ population in the specific context of the current study, minority groups may be less inclined to adopt embodied and practical identities of the broader, Dutch culture [44] but are more likely to blend and adapt influences from a variety of sources.

Other mediating factors may underlie the ethnic differences in body size ideals and body satisfaction. For example, the perception of a ‘normal’ body size is likely to be influenced by exposure within one’s social environment [22, 23, 45], thus greater prevalence of overweight/obesity within the African-origin populations may, in and of itself, drive the perceptions observed in this study. Moreover, future research should investigate how a broader range of factors such as cultural identity, importance of body ideals, socioeconomic indicators and concepts of culture that influence ethnic differences in body size ideals as well as body satisfaction [46, 47]. While the hypothesis was that the acculturation proxies mediate the association between ethnicity and body size ideals and body satisfaction, future research could investigate the moderating role of acculturation within these associations [48].

This study is unique because it investigated the differences in body size ideals and body satisfaction of two African-origin populations with a different migration history living in the same setting. Strengths of this study were the large population-based sample. The figural scale used in this study was designed for a multi-ethnic population and was validated in a sample of urban African Americans [9], making it a suitable tool for measuring body size ideals and body satisfaction in multi-ethnic populations.

Limitations of this study includes the cross-sectional study design. Acculturation is a process that happens through time, thus a prospective design would be more appropriate. Furthermore, this study design does not allow us to investigate causality (e.g. does a higher BMI lead to less body size satisfaction or does body size satisfaction lead to a lower BMI). Second, our measures of acculturation may not have captured the complexity of this topic, although a range of relevant variables were included in this study. Furthermore, the study only included a single item to measure ethnic identity which may not capture the multifaceted nature of the concept. Third, response rates in the HELIUS study were relatively low (31–35% in the ethnic groups included in this study) and this may have resulted in selection bias. However, all social-economic levels are represented and non-response analyses show that socio-economic differences between participants and non-participants were very small [17]. Fourth, the concept of body image is broader than what can be represented visually through Figure Rating scales and encompasses, amongst others, cognitions and emotions regarding one’s body and appearance. We were unable to include more detailed measures of body image in this study [49]. However, a figural scale is likely to be sufficient for the purpose of identifying population-level differences in body size preference in order to inform obesity prevention strategies. Finally, our definition of body satisfaction may have been too stringent; it is possible that participants with a body discrepancy score of 1 still feel satisfied with their current body.

In conclusion, African-origin participants had larger body size ideals, and, among those with overweight/obesity, were more likely to be satisfied with their body size than Dutch-origin participants. Furthermore, most women preferred a smaller body size, which may be cause for concern in Dutch women, where almost half preferred a smaller figure despite having a healthy weight status. Public health strategies promoting healthy behaviour and healthy weight should be cognisant of the need to target those at risk in order to reduce health inequalities. Lastly, factors other than acculturation may underlie ethnic differences in body size ideals and body satisfaction.

## Supporting information

S1 Checklist (DOCX)

S1 Fig The mediation model: Acculturation proxies mediate the relation between ethnicity and body size ideals or body satisfaction. (PDF)

S1 Table Measured BMI of participants according to figure selected to represent current body size. (DOCX)

S2 Table Ethnic differences in body satisfaction (satisfied versus prefer smaller) by weight status (n = 9819). (DOCX)
