# Supplementary figures and images for: Body size ideals and body satisfaction among Dutch-origin and African-origin residents of Amsterdam: The HELIUS study
Source: PLoS One. 2021 May 26;16(5):e0252054. doi: 10.1371/journal.pone.0252054 (PMC8153493; doi:10.1371/journal.pone.0252054)

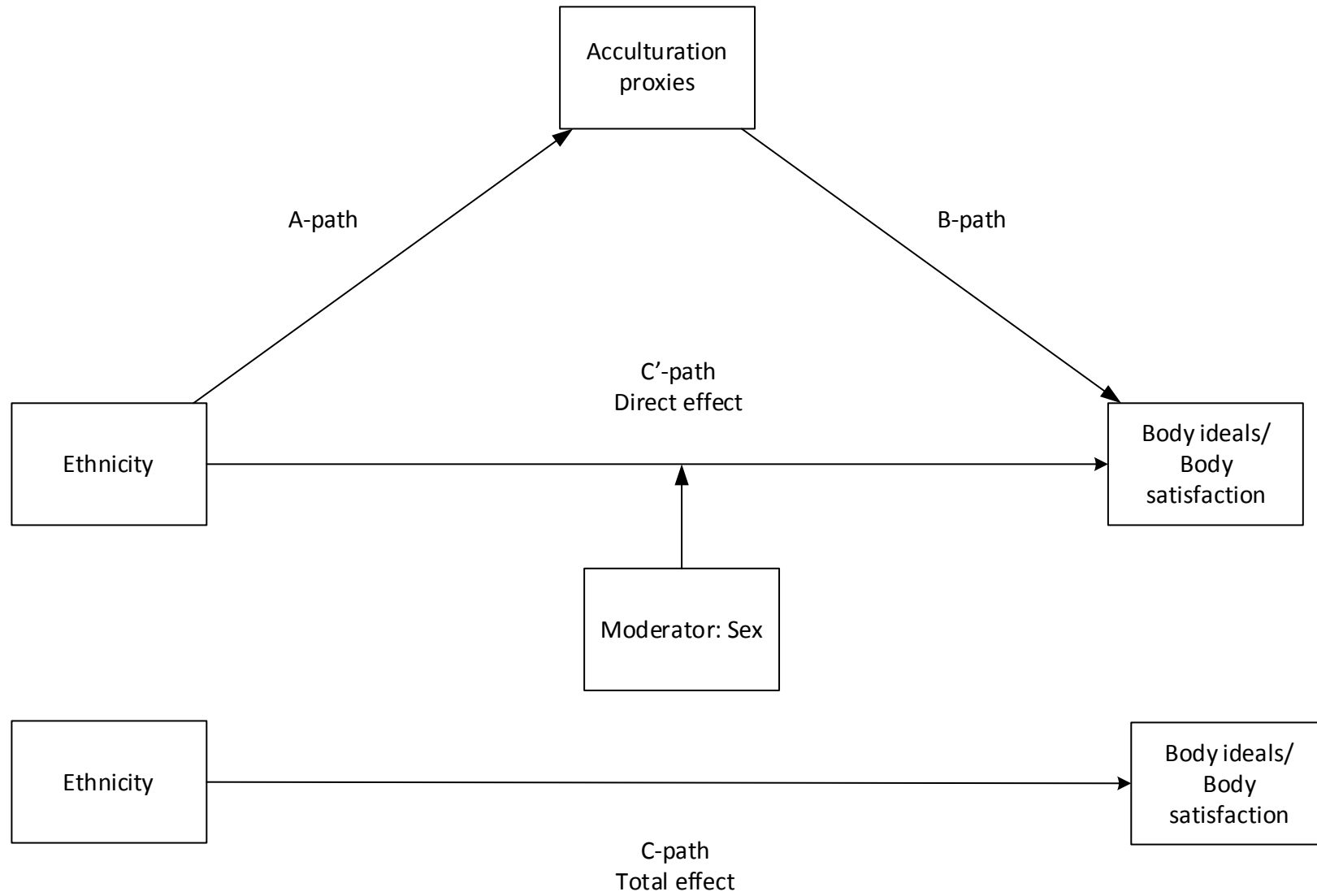

Supplement: S1 Fig — (PDF) [file pone.0252054.s002.pdf]
