# Supplementary material for: Body size ideals and body satisfaction among Dutch-origin and African-origin residents of Amsterdam: The HELIUS study
Source: PLoS One. 2021 May 26;16(5):e0252054. doi: 10.1371/journal.pone.0252054 (PMC8153493; doi:10.1371/journal.pone.0252054)
Supplement: S1 Table — (DOCX) [file pone.0252054.s003.docx]

S1 Table: Measured BMI of participants according to figure selected to represent current body size (n=10,854).

|  |  |  | Men |  |  | Women |  |
| --- | --- | --- | --- | --- | --- | --- | --- |
|  |  | Dutch  (n=2075) | Surinamese (n=1579) | Ghanaians (n=880) | Dutch (n=2456) | Surinamese (n=2476) | Ghanaians (n=1388) |
| 1 | N participants (%) | 24  (1.2%) | 29  (1.8%) | 12  (1.4%) | 37  (1.5%) | 39  (1.6%) | 12  (0.9%) |
|  | Mean BMI (SD) | 19.9  (2.5) | 21.4  (3.3) | 22.5  (4.1) | 18.8  (1.5) | 20.7  (3.6) | 21.5  (6.1) |
| 2 | N participants (%) | 242 (11.7%) | 153  (9.7%) | 46  (5.2%) | 312  (12.7%) | 191  (7.7%) | 70  (5.0%) |
|  | Mean BMI (SD) | 21.4  (1.9) | 21.7  (2.4) | 22.7  (2.4) | 20.1  (1.5) | 21.5  (2.4) | 22.2  (3.0) |
| 3 | N participants (%) | 452 (21.8%) | 400  (25.3%) | 188  (21.4%) | 712  (29.0%) | 404  (16.3%) | 200  (14.4%) |
|  | Mean BMI (SD) | 22.9  (1.9) | 23.8  (2.5) | 24.4  (2.4) | 21.9  (1.8) | 23.9  (2.8) | 25.0  (3.4) |
| 4 | N participants (%) | 699 (33.7%) | 577  (36.5%) | 360  (40.9%) | 746  (30.4%) | 632  (25.5%) | 335  (24.1%) |
|  | Mean BMI (SD) | 25.0  (2.1) | 26.4  (2.9) | 26.2  (2.7) | 24.3  (2.4) | 26.9  (3.2) | 27.5  (3.6) |
| 5 | N participants (%) | 550 (26.5%) | 342  (21.7%) | 199  (22.6%) | 392  (16.0%) | 565  (22.8%) | 322  (23.2%) |
|  | Mean BMI (SD) | 27.9  (3.0) | 29.9  (3.7) | 29.0  (2.7) | 27.4  (2.9) | 30.1  (3.5) | 29.9  (3.0) |
| 6 | N participants (%) | 72  (3.5%) | 53  (3.4%) | 46  (5.2%) | 143  (5.8%) | 303  (12.2%) | 232  (16.7%) |
|  | Mean BMI (SD) | 31.6  (4.0) | 33.3  (4.9) | 31.4  (3.4) | 30.9  (3.6) | 33.1  (4.2) | 32.8  (3.5) |
| 7 | N participants (%) | 28 (1.3%) | 15  (0.9%) | 27  (3.1%) | 74  (3.0%) | 211  (8.5%) | 162  (11.7%) |
|  | Mean BMI (SD) | 33.4  (3.8) | 34.2  (4.7) | 32.8  (6.2) | 33.0  (4.4) | 35.3  (4.5) | 35.0  (4.5) |
| 8 | N participants (%) | 4  (0.2%) | 10  (0.6%) | 1  (0.1%) | 29  (1.2%) | 111  (4.5%) | 47  (3.4%) |
|  | Mean BMI (SD) | 42.6  (7.6) | 39.0  (6.5) | 42.8  NA | 36.5  (5.1) | 38.9  (5.5) | 37.4  (5.1) |
| 9 | N participants (%) | 4  (0.2%) | - | 1  (0.1%) | 11  (0.4%) | 20  (0.8%) | 8  (0.6%) |
|  | Mean BMI (SD) | 33.9  (10.7) | - | 37.9  NA | 40.7  (6.8) | 43.8  (6.3) | 44.7  (6.6) |

Abbreviations: BMI= body mass index; SD= Standard Deviation
