# Supplementary material for: Body size ideals and body satisfaction among Dutch-origin and African-origin residents of Amsterdam: The HELIUS study
Source: PLoS One. 2021 May 26;16(5):e0252054. doi: 10.1371/journal.pone.0252054 (PMC8153493; doi:10.1371/journal.pone.0252054)
Supplement: S2 Table — (DOCX) [file pone.0252054.s004.docx]

S2 Table: Ethnic differences in body satisfaction (satisfied versus prefer smaller) by weight status (n=9819)

|  | Satisfied vs. prefer smaller | | | | | |
| --- | --- | --- | --- | --- | --- | --- |
|  | Men | | | Women | | |
|  | Dutch | Surinamese | Ghanaian | Dutch | Surinamese | Ghanaian |
| BMI |  | OR (95%CI) | OR (95%CI) |  | OR (95%CI) | OR (95%CI) |
| <24.99 | Ref | 2.81 (1.95;4.05) | 2.07 (1.29; 3.34) | Ref | 1.47 (1.12; 1.81) | 3.31 (2.18; 5,03) |
| >= 25.00 | Ref | 2.28 (1.86; 2.79) | 2.39 (1.88; 3.05) | Ref | 1.82 (1.42; 2.39) | 2.64 (1.98; 3.51) |

Adjusted for age and educational level

Abbreviations: OR= Odds Ratio, 95%CI= 95% Confidence Interval, Ref= reference group
