# Supplementary material for: Body size ideals and body satisfaction among Dutch-origin and African-origin residents of Amsterdam: The HELIUS study
Source: PLoS One. 2021 May 26;16(5):e0252054. doi: 10.1371/journal.pone.0252054 (PMC8153493; doi:10.1371/journal.pone.0252054)
Supplement: Peer review history

# Peer review: Decision Letter 0

**DOI:** 10.1371/journal.pone.0252054.r001

**Authors:** Frank T. Spradley

13 Apr 2021

PONE-D-21-06774

Body size ideals and body satisfaction among Dutch-origin and African-origin residents of  Amsterdam: The HELIUS Study

PLOS ONE

Dear Dr. Hoenink,

Thank you for submitting your manuscript to PLOS ONE. After careful consideration, we feel that it has merit but does not fully meet PLOS ONE’s publication criteria as it currently stands. Therefore, we invite you to submit a revised version of the manuscript that addresses the points raised during the review process.

Please submit your revised manuscript by May 28 2021 11:59PM. If you will need more time than this to complete your revisions, please reply to this message or contact the journal office at plosone@plos.org. When you're ready to submit your revision, log on to https://www.editorialmanager.com/pone/ and select the 'Submissions Needing Revision' folder to locate your manuscript file.

Please include the following items when submitting your revised manuscript:

- A rebuttal letter that responds to each point raised by the academic editor and reviewer(s). You should upload this letter as a separate file labeled 'Response to Reviewers'.
- A marked-up copy of your manuscript that highlights changes made to the original version. You should upload this as a separate file labeled 'Revised Manuscript with Track Changes'.
- An unmarked version of your revised paper without tracked changes. You should upload this as a separate file labeled 'Manuscript'.

If you would like to make changes to your financial disclosure, please include your updated statement in your cover letter. Guidelines for resubmitting your figure files are available below the reviewer comments at the end of this letter.

If applicable, we recommend that you deposit your laboratory protocols in protocols.io to enhance the reproducibility of your results. Protocols.io assigns your protocol its own identifier (DOI) so that it can be cited independently in the future. For instructions see: http://journals.plos.org/plosone/s/submission-guidelines#loc-laboratory-protocols. Additionally, PLOS ONE offers an option for publishing peer-reviewed Lab Protocol articles, which describe protocols hosted on protocols.io. Read more information on sharing protocols at https://plos.org/protocols?utm_medium=editorial-email&utm_source=authorletters&utm_campaign=protocols.

We look forward to receiving your revised manuscript.

Kind regards,

Frank T. Spradley

Academic Editor

PLOS ONE

Journal requirements:

When submitting your revision, we need you to address these additional requirements.

1. Please ensure that your manuscript meets PLOS ONE's style requirements, including those for file naming. The PLOS ONE style templates can be found at

https://journals.plos.org/plosone/s/file?id=wjVg/PLOSOne_formatting_sample_main_body.pdf and

https://journals.plos.org/plosone/s/file?id=ba62/PLOSOne_formatting_sample_title_authors_affiliations.pdf

2. Please provide additional details regarding participant consent. In the ethics statement in the Methods and online submission information, please ensure that you have specified (1) whether consent was informed and (2) what type you obtained (for instance, written or verbal, and if verbal, how it was documented and witnessed). If your study included minors, state whether you obtained consent from parents or guardians. If the need for consent was waived by the ethics committee, please include this information.

If you are reporting a retrospective study of medical records or archived samples, please ensure that you have discussed whether all data were fully anonymized before you accessed them and/or whether the IRB or ethics committee waived the requirement for informed consent. If patients provided informed written consent to have data from their medical records used in research, please include this information.

3. lease note that according to our submission guidelines (http://journals.plos.org/plosone/s/submission-guidelines), outmoded terms and potentially stigmatizing labels should be changed to more current, acceptable terminology. For example: “Caucasian” should be changed to “white” or “of [Western] European descent” (as appropriate).

4. We note that the grant information you provided in the ‘Funding Information’ and ‘Financial Disclosure’ sections do not match.

When you resubmit, please ensure that you provide the correct grant numbers for the awards you received for your study in the ‘Funding Information’ section.

5. We note that you have indicated that data from this study are available upon request. PLOS only allows data to be available upon request if there are legal or ethical restrictions on sharing data publicly. For information on unacceptable data access restrictions, please see http://journals.plos.org/plosone/s/data-availability#loc-unacceptable-data-access-restrictions.

In your revised cover letter, please address the following prompts:

a) If there are ethical or legal restrictions on sharing a de-identified data set, please explain them in detail (e.g., data contain potentially identifying or sensitive patient information) and who has imposed them (e.g., an ethics committee). Please also provide contact information for a data access committee, ethics committee, or other institutional body to which data requests may be sent.

b) If there are no restrictions, please upload the minimal anonymized data set necessary to replicate your study findings as either Supporting Information files or to a stable, public repository and provide us with the relevant URLs, DOIs, or accession numbers. Please see http://www.bmj.com/content/340/bmj.c181.long for guidelines on how to de-identify and prepare clinical data for publication. For a list of acceptable repositories, please see http://journals.plos.org/plosone/s/data-availability#loc-recommended-repositories.

We will update your Data Availability statement on your behalf to reflect the information you provide.

6. Your ethics statement should only appear in the Methods section of your manuscript. If your ethics statement is written in any section besides the Methods, please delete it from any other section.

Reviewers' comments:

Reviewer's Responses to Questions

**Comments to the Author**

1. Is the manuscript technically sound, and do the data support the conclusions?

The manuscript must describe a technically sound piece of scientific research with data that supports the conclusions. Experiments must have been conducted rigorously, with appropriate controls, replication, and sample sizes. The conclusions must be drawn appropriately based on the data presented. 

Reviewer #1: Yes

**********

2. Has the statistical analysis been performed appropriately and rigorously? 

Reviewer #1: Yes

**********

3. Have the authors made all data underlying the findings in their manuscript fully available?

The PLOS Data policy requires authors to make all data underlying the findings described in their manuscript fully available without restriction, with rare exception (please refer to the Data Availability Statement in the manuscript PDF file). The data should be provided as part of the manuscript or its supporting information, or deposited to a public repository. For example, in addition to summary statistics, the data points behind means, medians and variance measures should be available. If there are restrictions on publicly sharing data—e.g. participant privacy or use of data from a third party—those must be specified.

Reviewer #1: Yes

**********

4. Is the manuscript presented in an intelligible fashion and written in standard English?

PLOS ONE does not copyedit accepted manuscripts, so the language in submitted articles must be clear, correct, and unambiguous. Any typographical or grammatical errors should be corrected at revision, so please note any specific errors here.

Reviewer #1: Yes

**********

5. Review Comments to the Author

Please use the space provided to explain your answers to the questions above. You may also include additional comments for the author, including concerns about dual publication, research ethics, or publication ethics. (Please upload your review as an attachment if it exceeds 20,000 characters)

Reviewer #1: This is a very interesting study comparing body size ideals and body satisfaction in Dutch people compared to two migrant groups (Surinamese and Ghanaians) living in Amsterdam. This study has a large population-based sample size and uses a suitable tool for measuring body size ideals and body satisfaction in multi-ethnic populations. Participants had an average age of 43 years, a sample often underrepresented in research. The paper is well written and ideas are communicated clearly. This paper makes a unique contribution to the literature. Some aspects of the manuscript could be improved, following you find my suggestions:

1) Introduction: could you specify why there is a longer history of contact with Dutch culture for Surinamese people compared to Ghanaian people? It is explained in lines 123-129, I suggest to already explain it in the introduction.

Line 93, reference 16 does not talk about immigration.

2) Methods: Line 136-137- what about Ghanaian people? Were they included based on self-reported origin as well?

Line 197 – I do not think it is suitable to create only two categories (0= prefer smaller; 1= satisfied/prefer larger). There were 5% of Dutch, 19% of Surinamese and 33% of Ghanaian women who preferred to be larger, as well as 24% of Dutch, 33% of Surinamese and 37% of Ghanaian men. To put them in the same category as those who did not show discrepancies between their actual and ideal body size does not seem suitable and a lot of information is lost. Edwards, Tod & Molnar (2014) showed that there is a significant relationship between drive for muscularity and body dissatisfaction, which in turns means that body dissatisfaction might also be present when participants desire to be larger.

Line 197- a in “for those with a normal weight and overweight/obesity” is not needed

3) Results: In figure 1, there seem to be some data points missing for men in the body figures categories 8 & 9.

Line 270- Where is table S1?

4) Discussion: Line 460-461- Wouldn’t it be beneficial for everyone to promote healthy behaviours instead of promoting healthy weight, independently of ethnicity?

**********

6. PLOS authors have the option to publish the peer review history of their article (what does this mean?). If published, this will include your full peer review and any attached files.

If you choose “no”, your identity will remain anonymous but your review may still be made public.

**Do you want your identity to be public for this peer review?** For information about this choice, including consent withdrawal, please see our Privacy Policy.

Reviewer #1: **Yes: **Fabienne Andres

[NOTE: If reviewer comments were submitted as an attachment file, they will be attached to this email and accessible via the submission site. Please log into your account, locate the manuscript record, and check for the action link "View Attachments". If this link does not appear, there are no attachment files.]

While revising your submission, please upload your figure files to the Preflight Analysis and Conversion Engine (PACE) digital diagnostic tool, https://pacev2.apexcovantage.com/. PACE helps ensure that figures meet PLOS requirements. To use PACE, you must first register as a user. Registration is free. Then, login and navigate to the UPLOAD tab, where you will find detailed instructions on how to use the tool. If you encounter any issues or have any questions when using PACE, please email PLOS at figures@plos.org. Please note that Supporting Information files do not need this step.

# Author comment: Author response to Decision Letter 0

**DOI:** 10.1371/journal.pone.0252054.r002

19 Apr 2021

Editor Comments to Author:

1. Please ensure that your manuscript meets PLOS ONE's style requirements, including those for file naming. The PLOS ONE style templates can be found at

https://journals.plos.org/plosone/s/file?id=wjVg/PLOSOne_formatting_sample_main_body.pdf and

https://journals.plos.org/plosone/s/file?id=ba62/PLOSOne_formatting_sample_title_authors_affiliations.pdf

>> The manuscript has been adjusted to fit PLOS ONE’s style requirements.

2. Please provide additional details regarding participant consent. In the ethics statement in the Methods and online submission information, please ensure that you have specified (1) whether consent was informed and (2) what type you obtained (for instance, written or verbal, and if verbal, how it was documented and witnessed). If your study included minors, state whether you obtained consent from parents or guardians. If the need for consent was waived by the ethics committee, please include this information.

If you are reporting a retrospective study of medical records or archived samples, please ensure that you have discussed whether all data were fully anonymized before you accessed them and/or whether the IRB or ethics committee waived the requirement for informed consent. If patients provided informed written consent to have data from their medical records used in research, please include this information.

>> Additional information to the ethics statement in the Methods section has been provided.

Methods page 6, lines 115-118; “Written informed consent was obtained from all participants before starting the study (18). The study complies with the Declaration of Helsinki and was approved by the Institutional Review Board of the Amsterdam Medical Centre.”

3. Please note that according to our submission guidelines (http://journals.plos.org/plosone/s/submission-guidelines), outmoded terms and potentially stigmatizing labels should be changed to more current, acceptable terminology. For example: “Caucasian” should be changed to “white” or “of [Western] European descent” (as appropriate).

>> The term Caucasian has been adapted to white throughout the manuscript

4. We note that the grant information you provided in the ‘Funding Information’ and ‘Financial Disclosure’ sections do not match.

When you resubmit, please ensure that you provide the correct grant numbers for the awards you received for your study in the ‘Funding Information’ section.

>> I have made sure that the grant numbers in the funding information are the same numbers used in the financial disclosure section. 

5. We note that you have indicated that data from this study are available upon request. PLOS only allows data to be available upon request if there are legal or ethical restrictions on sharing data publicly. For information on unacceptable data access restrictions, please see http://journals.plos.org/plosone/s/data-availability#loc-unacceptable-data-access-restrictions.

In your revised cover letter, please address the following prompts:

a) If there are ethical or legal restrictions on sharing a de-identified data set, please explain them in detail (e.g., data contain potentially identifying or sensitive patient information) and who has imposed them (e.g., an ethics committee). Please also provide contact information for a data access committee, ethics committee, or other institutional body to which data requests may be sent.

b) If there are no restrictions, please upload the minimal anonymized data set necessary to replicate your study findings as either Supporting Information files or to a stable, public repository and provide us with the relevant URLs, DOIs, or accession numbers. Please see http://www.bmj.com/content/340/bmj.c181.long for guidelines on how to de-identify and prepare clinical data for publication. For a list of acceptable repositories, please see http://journals.plos.org/plosone/s/data-availability#loc-recommended-repositories.

We will update your Data Availability statement on your behalf to reflect the information you provide.

>>Our data availability agreement is the following:

The HELIUS data are owned by the Amsterdam University Medical Centers, location AMC in Amsterdam, The Netherlands. Any researcher can request the data by submitting a proposal to the HELIUS Executive Board as outlined at http://www.heliusstudy.nl/en/researchers/collaboration, by email: heliuscoordinator@amsterdamumc.nl. The HELIUS Executive Board will check proposals for compatibility with the general objectives, ethical approvals and informed consent forms of the HELIUS study. There are no other restrictions to obtaining the data and all data requests will be processed in the same manner.

6. Your ethics statement should only appear in the Methods section of your manuscript. If your ethics statement is written in any section besides the Methods, please delete it from any other section.

>> The ethics statement has been removed from the declarations section. 

Reviewer comments to authors:

Reviewer #1: This is a very interesting study comparing body size ideals and body satisfaction in Dutch people compared to two migrant groups (Surinamese and Ghanaians) living in Amsterdam. This study has a large population-based sample size and uses a suitable tool for measuring body size ideals and body satisfaction in multi-ethnic populations. Participants had an average age of 43 years, a sample often underrepresented in research. The paper is well written and ideas are communicated clearly. This paper makes a unique contribution to the literature. Some aspects of the manuscript could be improved, following you find my suggestions:

>> We would like to thank the reviewer for their positive feedback. 

1) Introduction: 

A. Could you specify why there is a longer history of contact with Dutch culture for Surinamese people compared to Ghanaian people? It is explained in lines 123-129, I suggest to already explain it in the introduction.

>> We have moved lines 123-129 to the end of the introduction.

Introduction pages 4-5, lines 84-104: “This study examined body size ideals and body satisfaction in two SSA-origin groups living in Amsterdam, the Netherlands, compared to the Dutch-origin population. Specifically, this study investigated; 1) the body size ideals of Dutch, African-Surinamese and Ghanaian origin men and women, 2) whether the two SSA-origin populations are more likely to be satisfied with their actual body size at a higher level of BMI than the Dutch and to assess whether this differs for persons with overweight and obesity, and 3) if potential differences in the body size ideals and body satisfaction between the two SSA-origin populations are explained by differences in acculturation levels. African-Surinamese and Ghanaians were included in the study as the two African-origin migrant groups have a different history of contact with the Dutch culture and, presumably, different levels of acculturation to the Dutch culture. The Surinamese originated from West Africa and were sent as slaves to Surinam between the 16th and 19th century. As a group, they have a long history of contact with the Dutch, speak Dutch and have been living in the Netherlands for many decades. Ghanaians are the largest SSA migrant group in the Netherlands with, on average, a shorter residence duration; many migrated in the beginning of the 1990’s. The hypothesis is that because the African-Surinamese have a longer history of contact with the Dutch culture compared to the Ghanaians, their body size ideals would be situated between those of the Dutch (smallest body size ideals) and Ghanaians (largest body size ideals). Any differences in body size ideals and body satisfaction between African-Surinamese and Ghanaians may be explained by their different acculturation levels.”

Methods page 6, lines 122-125: “As described in the introduction, the two African-origin migrant groups have a different history of contact with the Dutch culture and, presumably, different levels of acculturation to the Dutch culture due to their different lengths of contact with the Dutch.”

B. Line 93, reference 16 does not talk about immigration.

>> Indeed, reference 16 was not appropriate within the sentence in line 93. The reference of Gradidge et al. has been removed. 

Introduction page 4, lines 81-83; “For example, a study found that SSA-migrant women living in the United Kingdom had a more negative body image perception and were more dissatisfied with their body compared to the SSA-origin women (16).”

2) Methods: 

A. Line 136-137- what about Ghanaian people? Were they included based on self-reported origin as well?

>> Ethnicity for Ghanaian people was defined based on the country of birth. Because in Suriname there are five large ethnic groups, only for the Surinamese we based their ethnic origin based on their self-reported ethnic origin in order to differentiate them. In this study we only included African-origin Surinamese participants.

Methods pages 6, lines 132-133; “Because the Surinamese population mainly consists of five large ethnic groups, African-origin Surinamese (hereafter referred to as Surinamese) were included based on self-reported ethnic origin.”

B. Line 197 – I do not think it is suitable to create only two categories (0= prefer smaller; 1= satisfied/prefer larger). There were 5% of Dutch, 19% of Surinamese and 33% of Ghanaian women who preferred to be larger, as well as 24% of Dutch, 33% of Surinamese and 37% of Ghanaian men. To put them in the same category as those who did not show discrepancies between their actual and ideal body size does not seem suitable and a lot of information is lost. Edwards, Tod & Molnar (2014) showed that there is a significant relationship between drive for muscularity and body dissatisfaction, which in turns means that body dissatisfaction might also be present when participants desire to be larger.

>> Indeed participants that were deemed to prefer a smaller or a larger body size can both be seen as being unsatisfied (because of different reasons). We also agree with the reviewer that by dichotomizing this variable we lose information. Instead of dichotomizing the body satisfaction variable, we could have chosen for an ordinal variable (dissatisfied prefer smaller, satisfied, dissatisfied prefer larger). However, this would complicate the mediation analyses as conducting mediation analysis with an ordinal outcome is not possible in most packages, and if it is possible, the outcomes are more difficult to interpret. Furthermore, given that the proportion of participants who are dissatisfied and prefer to be larger is rather small (i.e. between 3% and 9% for women and between 14% and 17% for men when not stratifying the results by weight status), the addition of participants who prefer to be larger to the group of participants who are ‘satisfied’ would not largely influence the results or conclusions. To demonstrate this point, we conducted logistic analyses and only compared those who prefer a smaller body with those who were satisfied. When comparing the results displayed in the table below to Table 3 in the manuscript, we find slightly smaller Odds Ratios when excluding participants who prefer to be larger from the satisfied group (e.g., an OR of 2.81 instead of 2.96 for Surinamese men with a normal weight). Nevertheless, all ORs are still significant and larger than 1, thereby resulting in the same conclusion as when we do include those who prefer a larger body. To demonstrate that the results and conclusions are not really influenced by combining the group of satisfied participants with participants who prefer to be larger, we have added the Table below to the supplementary file. Furthermore, from the hypothesis within this paper (that Surinamese and Ghanaians would want smaller body figures as their exposure to the Dutch culture increases) it made sense to focus on participants preferring to be smaller rather than larger. 

S2 Table: Ethnic differences in body satisfaction (satisfied versus prefer smaller) by weight status (n=9819)

 Satisfied vs. prefer smaller

 Men Women

 Dutch Surinamese Ghanaian Dutch Surinamese Ghanaian

BMI OR (95%CI) OR (95%CI) OR (95%CI) OR (95%CI)

<24.99 Ref 2.81 (1.95;4.05) 2.07 (1.29; 3.34) Ref 1.47 (1.12; 1.81) 3.31 (2.18; 5,03)

>= 25.00 Ref 2.28 (1.86; 2.79) 2.39 (1.88; 3.05) Ref 1.82 (1.42; 2.39) 2.64 (1.98; 3.51)

Adjusted for age and educational level

Abbreviations: OR= Odds Ratio, 95%CI= 95% Confidence Interval, Ref= reference group

Methods page 9, lines 195-202: “Logistic regression analysis instead of ordinal regression analysis was used for the outcome body satisfaction given the low proportion of participants being unsatisfied and preferring a larger body figure, the better interpretability of the results and the possibility to assess mediation by acculturation. As it can be argued that participants who are satisfied should not be combined with participants who prefer to be larger (as they are not satisfied with their body), we conducted a sensitivity analysis (0= prefer smaller; 1= satisfied) to assess whether the addition of those preferring a larger body affected the results in the main analyses.”

Results page 16, lines 301-303: “The same results, with slightly smaller effect sizes, were found when comparing participants who were satisfied with their body to those who preferred to be smaller (S2 Table).”

C. Line 197- a in “for those with a normal weight and overweight/obesity” is not needed

>> Because we stratify the results of the logistic regression analyses by weight status, we find that it is useful to specify this in the Methods section.

3) Results: 

A. In figure 1, there seem to be some data points missing for men in the body figures categories 8 & 9.

>> That is correct, as only one male or no male selected these body figures to represent their current body.

B. Line 270- Where is table S1?

>> S1 Table can be found in the supplementary material.

4) Discussion: 

A. Line 460-461- Wouldn’t it be beneficial for everyone to promote healthy behaviours instead of promoting healthy weight, independently of ethnicity

>> Indeed, it would be beneficial to promote healthy behaviours/weights independent of ethnicity. However, given the larger health problems among these migrants, special attention should be provided to these populations in order to not increase the ethnic inequalities. The sentence has been adapted as follows:

Discussion page 26, lines 471-473: “Public health strategies promoting healthy behaviour and healthy weight should be cognisant of the need to target those at risk in order to reduce health inequalities.”

Attachment Submitted filename: Response to Reviewers.docx Click here for additional data file.

# Peer review: Decision Letter 1

**DOI:** 10.1371/journal.pone.0252054.r003

**Authors:** Frank T. Spradley

10 May 2021

Body size ideals and body satisfaction among Dutch-origin and African-origin residents of  Amsterdam: The HELIUS Study

PONE-D-21-06774R1

Dear Dr. Hoenink,

We’re pleased to inform you that your manuscript has been judged scientifically suitable for publication and will be formally accepted for publication once it meets all outstanding technical requirements.

Within one week, you’ll receive an e-mail detailing the required amendments. When these have been addressed, you’ll receive a formal acceptance letter and your manuscript will be scheduled for publication.

An invoice for payment will follow shortly after the formal acceptance. To ensure an efficient process, please log into Editorial Manager at http://www.editorialmanager.com/pone/, click the 'Update My Information' link at the top of the page, and double check that your user information is up-to-date. If you have any billing related questions, please contact our Author Billing department directly at authorbilling@plos.org.

If your institution or institutions have a press office, please notify them about your upcoming paper to help maximize its impact. If they’ll be preparing press materials, please inform our press team as soon as possible -- no later than 48 hours after receiving the formal acceptance. Your manuscript will remain under strict press embargo until 2 pm Eastern Time on the date of publication. For more information, please contact onepress@plos.org.

Kind regards,

Frank T. Spradley

Academic Editor

PLOS ONE

Reviewers' comments:

Reviewer's Responses to Questions

**Comments to the Author**

1. If the authors have adequately addressed your comments raised in a previous round of review and you feel that this manuscript is now acceptable for publication, you may indicate that here to bypass the “Comments to the Author” section, enter your conflict of interest statement in the “Confidential to Editor” section, and submit your "Accept" recommendation.

Reviewer #1: All comments have been addressed

**********

2. Is the manuscript technically sound, and do the data support the conclusions?

The manuscript must describe a technically sound piece of scientific research with data that supports the conclusions. Experiments must have been conducted rigorously, with appropriate controls, replication, and sample sizes. The conclusions must be drawn appropriately based on the data presented. 

Reviewer #1: Yes

**********

3. Has the statistical analysis been performed appropriately and rigorously? 

Reviewer #1: Yes

**********

4. Have the authors made all data underlying the findings in their manuscript fully available?

The PLOS Data policy requires authors to make all data underlying the findings described in their manuscript fully available without restriction, with rare exception (please refer to the Data Availability Statement in the manuscript PDF file). The data should be provided as part of the manuscript or its supporting information, or deposited to a public repository. For example, in addition to summary statistics, the data points behind means, medians and variance measures should be available. If there are restrictions on publicly sharing data—e.g. participant privacy or use of data from a third party—those must be specified.

Reviewer #1: Yes

**********

5. Is the manuscript presented in an intelligible fashion and written in standard English?

PLOS ONE does not copyedit accepted manuscripts, so the language in submitted articles must be clear, correct, and unambiguous. Any typographical or grammatical errors should be corrected at revision, so please note any specific errors here.

Reviewer #1: Yes

**********

6. Review Comments to the Author

Please use the space provided to explain your answers to the questions above. You may also include additional comments for the author, including concerns about dual publication, research ethics, or publication ethics. (Please upload your review as an attachment if it exceeds 20,000 characters)

Reviewer #1: All my comments were fully addressed. I enjoyed reading your paper and believe it makes a valuable contribution to the literature. I wish you all the best.

**********

7. PLOS authors have the option to publish the peer review history of their article (what does this mean?). If published, this will include your full peer review and any attached files.

If you choose “no”, your identity will remain anonymous but your review may still be made public.

**Do you want your identity to be public for this peer review?** For information about this choice, including consent withdrawal, please see our Privacy Policy.

Reviewer #1: **Yes: **Fabienne Andres

# Editor report: Acceptance letter

**DOI:** 10.1371/journal.pone.0252054.r004

**Authors:** Frank T. Spradley

12 May 2021

PONE-D-21-06774R1 

Body size ideals and body satisfaction among Dutch-origin and African-origin residents of  Amsterdam: The HELIUS Study 

Dear Dr. Hoenink:

I'm pleased to inform you that your manuscript has been deemed suitable for publication in PLOS ONE. Congratulations! Your manuscript is now with our production department. 

If your institution or institutions have a press office, please let them know about your upcoming paper now to help maximize its impact. If they'll be preparing press materials, please inform our press team within the next 48 hours. Your manuscript will remain under strict press embargo until 2 pm Eastern Time on the date of publication. For more information please contact onepress@plos.org.

If we can help with anything else, please email us at plosone@plos.org. 

Thank you for submitting your work to PLOS ONE and supporting open access. 

Kind regards, 

PLOS ONE Editorial Office Staff

on behalf of

Dr. Frank T. Spradley 

Academic Editor

PLOS ONE
